# The AMPK Agonist AICAR Inhibits TGF-β1 Induced Activation of Kidney Myofibroblasts

**DOI:** 10.1371/journal.pone.0106554

**Published:** 2014-09-04

**Authors:** Kuan-Hsing Chen, Hsiang-Hao Hsu, Cheng-Chia Lee, Tzu-Hai Yen, Yi-Ching Ko, Chih-Wei Yang, Cheng-Chieh Hung

**Affiliations:** Kidney Research Center, Chang Gung Memorial Hospital, Chang Gung University, School of Medicine, Taoyuan, Taiwan; Rouen University Hospital, France

## Abstract

Activation of interstitial myofibroblasts and excessive production of extracellular matrix proteins are common pathways that contribute to chronic kidney disease. In a number of tissues, AMP-activated kinase (AMPK) activation has been shown to inhibit fibrosis. Here, we examined the inhibitory effect of the AMPK activator, 5-aminoimidazole-4-carboxyamide ribonucleoside (AICAR), on renal fibrosis *in* *vivo* and TGF-β1-induced renal fibroblasts activation *in* *vitro*. A unilateral ureteral obstruction (UUO) model was induced in male BALB/c mice. Mice with UUO were administered AICAR (500 mg/Kg/day) or saline intraperitoneally 1 day before UUO surgery and daily thereafter. Both kidneys were harvested 7 days after surgery for further analysis. For the in vitro studies, NRK-49F rat fibroblasts were pre-incubated with AICAR before TGF-β1 stimulation. The inhibitory effects of AICAR on signaling pathways down-stream of TGF-β1 were analyzed. In UUO model mice, administration of AICAR attenuated extracellular matrix protein deposition and the expression of α-smooth muscle actin (α-SMA), type I collagen and fibronectin. Pre-incubation of NRK-49F cells with AICAR inhibited TGF-β1-induced myofibroblast activation. Silencing of AMPKα1 by siRNA or by blocking AMPK activation with Compound C diminished the inhibitory effect of AICAR. Moreover, the inhibitory effects of AICAR on TGF-β1-mediated myofibroblast activation were associated with down-regulation of ERK 1/2 and STAT3. Our results suggest that AICAR reduces tubulointerstitial fibrosis in UUO mice and inhibits TGF-β1-induced kidney myofibroblast activation. AMPK activation by AICAR may have therapeutic potential for the treatment of renal tubulointerstitial fibrosis.

## Introduction

Renal tubulointerstitial fibrosis which is characterized by aberrant activation and renal fibroblasts growths is a common pathway in end-stage renal disease [1], [2]. Excessive accumulation of extracellular matrix (ECM) proteins, such as type I and IV collagens, α-smooth muscle actin (α-SMA), and fibronectin is the hallmark of tubulointerstitial fibrosis [3]. In addition, differentiation of renal interstitial fibroblasts into α-SMA (+) myofibroblasts is the crucial step in the development of renal fibrosis. Therefore, targeting the signaling pathways that mediate fibroblast-myofibroblast transformation may attenuate the progression of renal fibrosis.

Transforming growth factor-β1 (TGF-β1) signaling is the most important pathway associated with renal fibroblast-myofibroblast activation [4]–[6]. TGF-β1 activates downstream Smads signaling, which phosphorylates the Smad2/Smad3 complex and translocates cytosolic Smad4 into the nucleus to regulate fibrosis-related gene expression [7], [8]. In addition to the canonical TGF-β/Smads pathway, TGF-β1 also induces myofibroblast activation and renal fibrosis through non-Smad signaling pathways, including mitogen-activated protein kinase (MAPK), PI3K-Akt, small GTPase pathways, and etc. [9], [10].

AMP-activated kinase (AMPK), which is a heterotrimeric protein consisting of α1/2-, β1/2-, and γ1/2/3- subunits, is a sensor of cellular energy status [11]. AMPK activation is dependent on Thr172 phosphorylation of the α-subunit [12]. AMPK has been shown to be activated by an increased intracellular AMP/ATP ratio [11]. In addition, recent studies have shown that AMPK can also be activated by AMP/ATP ratio independent pathways, such as activation of Ca2+/calmodulin-dependent protein kinase kinases [13], [14]. The biological effects of AMPK activation are diverse, and they include lipid and glucose metabolism [15], [16], regulation of cytokine production and inflammatory status [17], [18], and cellular proliferation and apoptosis [19], [20]. In addition, AMPK activation has been shown to inhibit the fibrogenic responses of hepatic stellate cells and has the potential to be a novel therapeutic target for liver fibrosis treatment [21]. The AMPK activator, 5-amino-4-imidazolecarboxamide riboside-1-β-D-ribofuranoside (AICAR) is an adenosine analogue, which is taken up by adenosine transporters on the cell membrane and is subsequently phosphorylated to generate ZMP. ZMP mimics AMP and stimulates AMPK phosphorylation in the cell [22]. AICAR was first reported as a regulator of cellular metabolism [23]. Subsequently, AICAR has been shown to have regulatory effects on diverse biological processes, including lipid and glucose metabolism [24], proinflammatory response [25], cytokine production [26], cell proliferation and apoptosis [27], [28]. In addition, activation of AMPK by AICAR has been shown to negatively regulate mesangial-myofibroblast transdifferentiation by TGF-β1 *in* *vitro* [29]. Recently, AICAR has also been shown to ameliorate ischemic/reperfusion injury [30] and kidney fibrosis in a rat subtotal nephrectomy model [31]. However, the mechanisms underlying the inhibitory effect of AICAR on TGF-β1-mediated renal fibroblast-myofibroblast transformation and renal fibrosis are not fully understood.

This study attempts to examine the anti-fibrotic effect of AMPK pathway activation by AICAR in both an *in* *vivo* model of tubulointerstitial fibrosis after unilateral ureteral obstruction (UUO) and an *in* *vitro* model of renal fibroblast.

## Materials and Methods

### Ethics statement

All animal experiments were carried out in accordance with the guidelines issued by the Animal Care and Ethics Committee on Research at Chang Gung Memorial Hospital. This study was approved by the Animal Care and Use Committee of Chang Gung Memorial Hospital (approval number 2010121411).

### Reagents

Bovine calf serum (BCS) and Dulbecco’s modified Eagle’s medium (DMEM), were purchased from HyClone Laboratories, Inc. (South Logan, UT, USA). AICAR and α-smooth muscle actin (SMA) antibody were obtained from Sigma Chemical Co. (St Louis, MO, USA). Phospho-specific AMPKα, ERK1/2, JNK1/2, Smad2, Smad3, JNK, p38, STAT3 antibodies were purchased from Cell Signaling Technology (Beverly, MA, USA). Collagen I and IV antibodies were obtained from Southern Biotech (Birmingham, AL). Fibronectin and β-actin antibodies were purchased from Abcam (Abcam, Cambridge, MA). All other chemicals were obtained from Sigma Chemical Co. Recombinant human TGF-β1 was purchased from R&D Systems (Minneapolis, MN, USA).

### Animal model of unilateral ureteral obstruction

Unilateral ureteral obstruction (UUO) was induced in adult male *BALB/c mice* (3–5 months old, 20–25 g) under 2.5% avertin-induced anesthesia. The left kidney and ureter were exposed via a flank incision, and then the ureter was ligated at two points proximal to the kidney with 6–0 silk. The wound was closed in layers. In sham animals, the kidney and ureter were exposed, but the ureter was not tied. Mice with UUO were administered intraperitoneally AICAR (500 mg/Kg body weight perday) or saline 1 day before the UUO surgery and daily thereafter. Both the obstructed and contralateral kidneys were harvested 7 days after surgery. The number of animals in each group was 6.

### Kidney tissue preparation

Mice were anesthetized, sacrificed and both kidneys were harvested. Kidneys were hemi-sectioned and portions were snap frozen in liquid nitrogen for real time-PCR or western blot analysis. Some kidneys were fixed in 10% neutral buffered formalin at 4°C for 12 h, processed, embedded in paraffin wax, sliced into 4 mm sections, and stored at room temperature until use.

### Renal fibrosis analysis

To assess renal fibrosis, Gomori’s trichrome staining was performed according to the manufacturer’s instructions (Leica Biosystems Richmond, Inc., Richmond, IL, USA). Ten individual high-power fields (magnification, 200×) per kidney were analyzed. The percentage area occupied by collagen tissue (blue color) was analyzed by using computer-assisted image analysis software (MetaMorph, version 4.6, Universal Imaging Corporation, Downingtown, PA, USA).

### Immunofluorescence and immunohistochemical staining

Paraffin sections (4 µm) of renal tissue were analyzed by immunohistochemistry using monoclonal antibodies against α-SMA (1∶500), collagen I (1∶500), and fibronectin (1∶500). To detect collagen I and fibronectin immunostaining of the sections, a biotinylated secondary antibody (1∶200, incubated for 1 h at 37°C) and streptavidin–biotin–peroxidase (1∶200, incubated for 1 h at 37°C) were used. The sections were then counterstained with hematoxylin, dehydrated and mounted. To detect α-SMA immunofluorescence staining, the sections were incubated with fluorescent-conjugated secondary antibodies (1∶500). For each kidney, ten individual high-power fields (magnification, 200×) per kidney were analyzed and representative images are presented.

### Real time-PCR

Real time-PCR was performed using total RNA isolated from the kidney samples in an ABI-Prism 7000 with SYBR Green I as a double-stranded DNA-specific dye, according to the manufacturer’s instructions (PE-Applied Biosystems, Cheshire, UK). The expression of 18S mRNA was used as an internal control. Sequences of the primers used are listed in Table 1. Primers were constructed such that they were compatible with a single RT-PCR thermal profile (95°C for 10 min, and 40 cycles of 95°C for 30 s and 60°C for 1 min). The change in gene expression was determined for the control, UUO, and UUO + AICAR treated mice.

**Table 1:** Forward and reverse primers used for quantitative real-time reverse transcriptase PCR.

Gene	Type	Sequences (5′→3′)
Collagen I	Forward	AAGGGGTCTTCCTGGTGAAT
	Reverse	GGGGTACCACGTTCTCCTC
Fibronectin	Forward	TGTGACCAGCAACACGGTG
	Reverse	ACAACAGGAGAGTAGGGCGC
TGF-β1	Forward	TGCGTCTGCTGAGGCTCAA
	Reverse	TTGCTGAGGTATCGCCAGGA
TNF-α		Mm99999068_m1
MCP-1		Mm00441242_m1
18S rRNA		Hs99999901_s1

Abbreviations: Collagen I, collagen type-I; TGF-β1, transforming growth factor-β1; TNF, tumor necrosis factor; MCP-1, monocyte chemotactic protein-1.

### Cell culture and experimental treatments

NRK-49F rat fibroblasts (ATCC, Manassas, VA, USA) were grown in DMEM containing 5% BCS at 37°C in a 5% CO_2_ atmosphere. Cells were seeded and allowed to adhere overnight, and then the medium was changed to DMEM containing 0.1% FBS. Cells were stimulated with recombinant human TGF-β1 (1 ng/mL) for the indicated periods before harvesting. Alternatively, NRK-49F cells were pre-incubated with AICAR or specific inhibitors for 30 mins before TGF-β1 treatment. Total RNA was extracted for real-time PCR and total cell lysates were extracted for western blot analysis. All measurements were performed at least in triplicate.

### Small interfering RNA (siRNA)

siRNAs were synthesized and constructed according to the manufacturer’s protocol (siGENOME and ON-TARGET*plus;* Thermo Fisher Scientific., Lafayette, CO, USA.). The following targeting sequences were used: ON-TARGET*plus* SMARTpool for rat AMPKα1 (catalog number: L-091373-00-0010) and rat AMPKα2 (catalog number: L-100623-00-0010). Transfection agent with a scrambled siRNA (Dharmacon RNAi control; Thermo Fisher Scientific) served as a control. Cells were seeded into 6-well plates at a concentration of 1×10^5^ cells/well and incubated for 24 h before transfection. Cells were transfected with siRNAs in serum-free DMEM using DharmaFECT1 (Thermo Fisher Scientific) according to the manufacturer’s protocol. After 24 h of incubation, the transfected cells were serum-deprived for 8 h, and then used in the following experiments.

### Western blot analysis

Total cellular protein was extracted as described previously [32]. Equal amounts of proteins were mixed with an equal volume of reducing SDS sample buffer and boiled at 95°C for 5 mins. Protein samples were resolved by 10% SDS-PAGE and then electroblotted on nitrocellulose membranes (Bio-Rad, Hercules, CA). After electroblotting, nonspecific binding was blocked with 5% nonfat milk. The membrane was incubated with primary antibodies overnight at 4°C, and then incubated with horseradish peroxidase–conjugated secondary antibodies for 1 h at room temperature. The primary antibodies against the following proteins were used at 1∶1,000 dilutions unless otherwise indicated: collagen I, collagen IV, α-SMA, AMPK-α, phospho-AMPK-α (Thr172), phospho-Smad2, phospho-Smad3, phopho-ERK1/2, phospho-p38, phospho-JNK, phospho-STAT3 (Tyr-705), anti-tubulin (1∶20,000), and anti-β-actin (1∶20,000). Immunoreactive bands were visualized by enhanced chemiluminescence (Amersham, Arlington Heights, IL, USA) as described previously [33].

### Statistical analysis

All the experiments were conducted at least three times. Data depicted in graphs represent the mean ± S.E. for each group. One-way analysis of variance (ANOVA) was conducted for multiple-group comparisons, and Tukey’s post-hoc analysis was used to evaluate the significance of paired groups in the animal study. In the NRK-49F rat fibroblasts study, Student’s t test was used to calculate statistical significance between different conditions. In all analyses, p values less than 0.05 were considered statistically significant.

## Results

### Administration of AICAR inhibited kidney fibrosis in UUO mice

**Figure 1:**
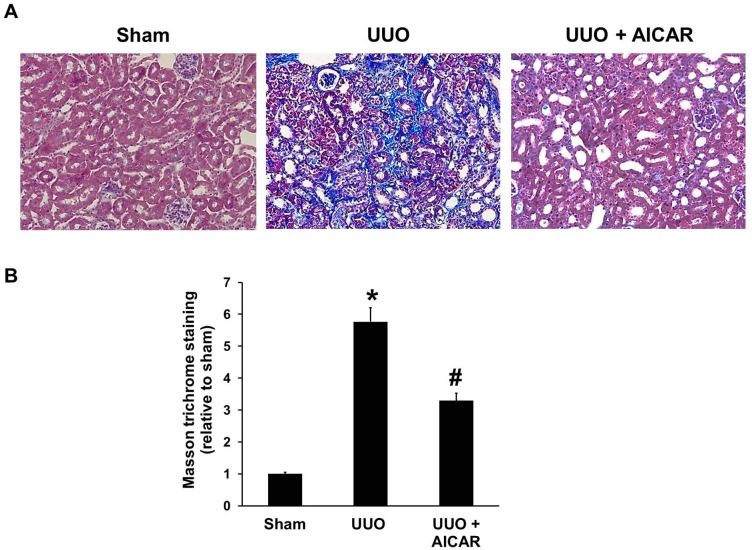
AICAR reduces renal fibrosis and the deposition of extracellular matrix (ECM) in obstructed kidneys. A unilateral ureteral obstruction (UUO) model was induced in adult male BALB/c mice. Sham-operated animals had their kidney exposed but the ureter was not tied. Mice with UUO were administered intra-peritoneal AICAR (500 mg·kg^−1^·day) or saline 1 day before the UUO surgery and daily thereafter. Obstructed kidneys were harvested 7 days after surgery. (A) Photomicrographs illustrating Masson trichrome staining of kidney tissue from mice in various treatment groups. (B) The Masson trichrome-positive tubulointerstitial area (blue) relative to the total area from 10 random cortical fields was analyzed. Data are shown as the mean ± S.E. (n = 6 in each group). *P<0.05 versus the sham group; #*P*<0.05 versus the UUO group.

No morphological, cellular, or molecular differences were observed in the non-obstructed contralateral kidneys from mice in both experimental groups (UUO + saline and UUO + AICAR; data not shown). The mean serum levels of blood urea nitrogen (BUN, mg/dL) was 18.3±0.95 in sham animals, whereas BUN serum levels in UUO + saline and UUO + AICAR mice were 21.67±0.66 and 21.17±1.22, respectively, which were not significant different from each other. Compared with the sham control kidneys (Fig. 1A, left panel), kidneys from mice with UUO developed a severe tubulointerstitial injury consisting of marked tubular dilatation and atrophy, interstitial inflammation and fibrosis (Fig. 1A, middle panel). Glomeruli and vessels were well preserved. Masson’s trichrome staining revealed intense deposition of extracellular matrix in the tubuloinsterstitium of obstructed kidneys. Administration of AICAR significantly attenuated extracellular matrix deposition (Fig. 1A, right panel). Renal fibrosis analysis showed that AICAR attenuated UUO-induced tubulointerstitial fibrosis compared with that in the UUO + saline group (Fig. 1B).

**Figure 2:**
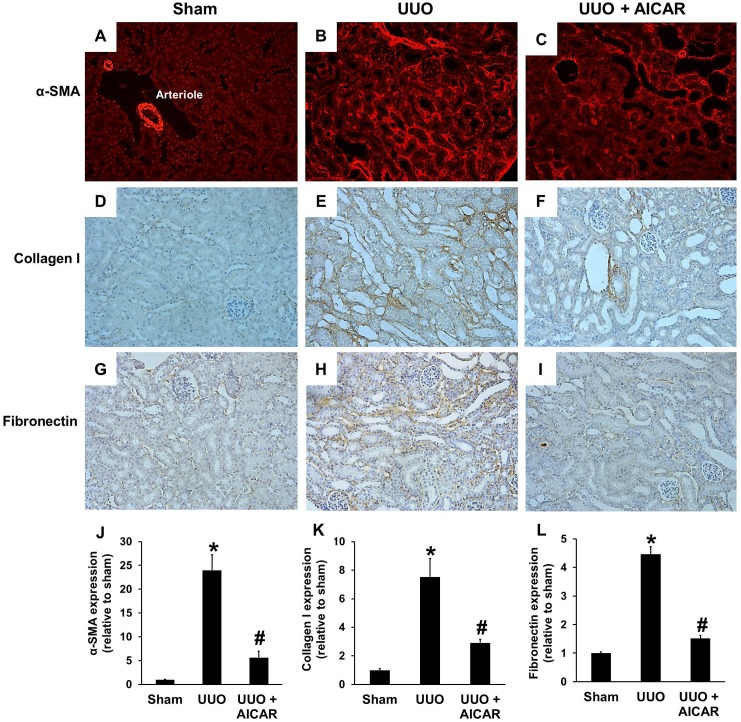
AICAR reduces the expression of α-smooth muscle actin (α-SMA), collagen I, and fibronectin. A unilateral ureteral obstruction (UUO) model was induced in adult male BALB/c mice. Sham-operated animals had their kidney exposed but the ureter was not tied. Mice with UUO were administered intra-peritoneal AICAR (500 mg·kg^−1^·day) or saline 1 day before the UUO surgery and daily thereafter. Obstructed kidneys were harvested 7 days after surgery. Tubulointerstitial expression of α-SMA (A, B, C, J), collagen I (D, E, F, K) and fibronectin (G, H, I, L) in sham, UUO, and UUO + AICAR groups were evaluated by immunofluorescence and immunohistochemistry analysis. Each panel is a randomly acquired representative image of the cortex area in one of six animals (magnification, 200×). The positive staining area relative to the total area from 10 random cortical fields was analyzed. Data are shown as the mean ± S.E. (n = 6 in each group) in the bar graph. *P<0.05 versus the sham group; #*P*<0.05 versus the UUO group.

α-SMA, a marker of kidney myofibroblast activation, was assessed by immunofluorescence staining. Kidneys without ureteral obstruction normally express αSMA around the renal arterioles (Fig. 2A). Seven days after UUO, αSMA expression in the tubulointerstitium of the obstructed kidney was significantly increased (Fig. 2B). AICAR administration significantly attenuated myofibroblast activation in the obstructed kidney, which presented as reduced α-SMA expression (Fig. 2C). Another marker of myofibroblast activation, type I collagen, was also evaluated by immunohistochemical staining. As shown in Figure 2D, basal levels of type I collagen expression were detected in the kidney of sham-operated mice. UUO injury led to a significant increase in collagen I expression (Fig. 2E). AICAR treatment decreased collagen I expression to near the basal level in the obstructed kidney (Fig. 2F). Fibronectin expression was also assessed to determine whether AICAR administration inhibits the expression of other ECM proteins. Fibronectin immunohistochemical staining showed increased expression of fibronectin after UUO injury (Fig. 2H), and fibronectin expression was decreased by AICAR administration (Fig. 2I). The expression of phospho-AMPK was increased after administration of AICAR in UUO mice, compared to that of sham and UUO group (Figure S1).

**Figure 3:**
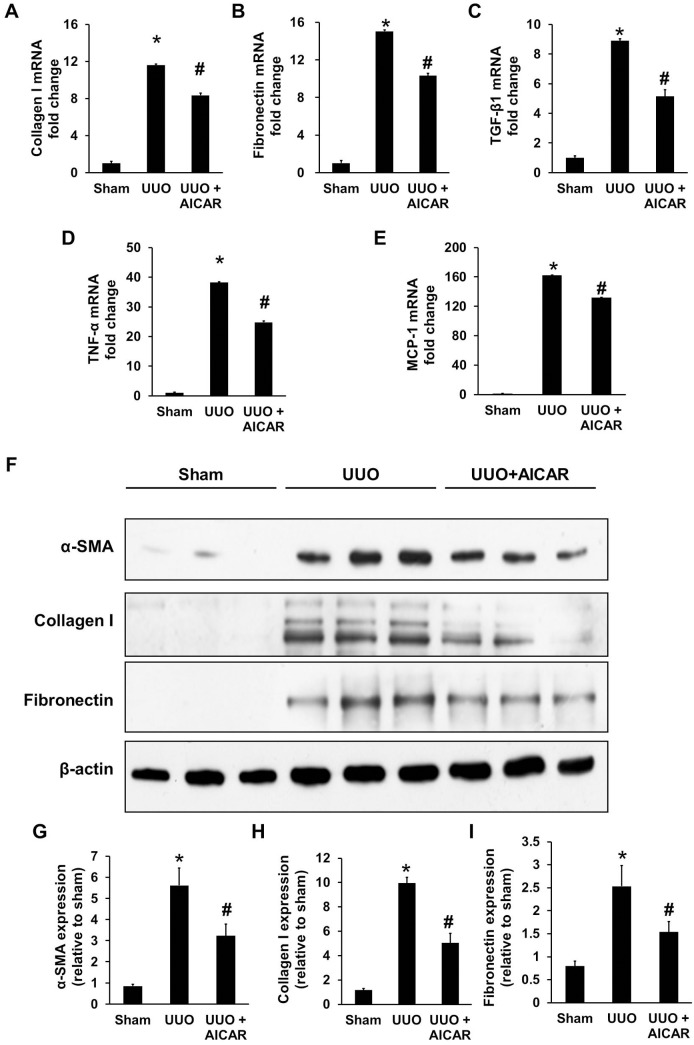
AICAR reduces the expression of α-smooth muscle actin (α-SMA), collagen I, and fibronectin. A unilateral ureteral obstruction (UUO) model was induced in adult male BALB/c mice. Sham animals had their kidney exposed but the ureter was not tied. Mice with UUO were administered intra-peritoneal AICAR (500 mg·kg^−1^·day) or saline 1 day before the UUO surgery and daily thereafter. Obstructed kidneys were harvested 7 days after surgery. qPCR analysis of the mRNA expression of collagen I (A), fibronectin (B), TGF-β1 (C), TNF-α (D) and MCP-1 (E), in sham, UUO, and UUO + AICAR kidneys. Data are expressed relative to the expression in sham-operated kidneys. Kidney tissue lysates were also subjected to immunoblot analysis with specific antibodies against α-SMA, collagen I, fibronectin, and β-actin (F). Protein expression levels of α-SMA (G), collagen I (H) and fibronectin (I) were analyzed by western blotting, quantified by densitometry, and normalized to β-actin levels. Each bar represents the mean ± S.E. (n = 6 in each group). *P<0.05 between the sham and UUO group; #*P*<0.05 between the UUO and UUO + AICAR group.

The mRNA expression of type I collagen, fibronectin and TGF-β1 was analyzed in kidney tissue lysates. RT-PCR analysis revealed that UUO injury significantly increased the expression of type I collagen, fibronectin and TGF-β1, whereas AICAR treatment attenuated this increased expression (Fig. 3A, 3B and 3C, respectively). AICAR treatment also significantly reduced the increased expression of the inflammatory cytokines TNF-α and MCP-1 in UUO mice (Fig. 3D, E). In agreement with the mRNA expression results, western blotting also showed that AICAR had a similar inhibitory effect on protein levels (Fig. 3F). The bar graphs of the protein levels of α-SMA, collagen I, and fibronectin are shown in Figures 3G, 3H, and 3I, respectively.

Taken together, these data show that AICAR treatment significantly attenuated myofibroblast activation and ECM proteins deposition in UUO mice.

### TGF-β1 activation of fibroblast-myofibroblast transformation is inhibited by AICAR

**Figure 4:**
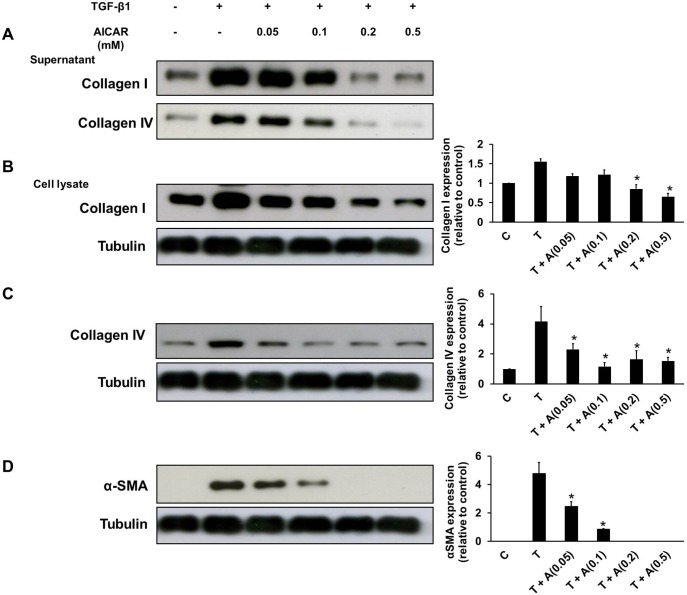
AICAR inhibits transforming growth factor-β1 (TGF-β1)-induced fibroblast-myofibroblast transformation. Cultured rat fibroblasts, NRK-49F cells were pre-incubated in the presence or absence of AICAR (0.05 mM–0.5 mM) for 30 mins. Then, these cells were stimulated with TGF-β1 (1 ng/mL) for 24 h before harvesting. The culture supernatant was subject to immunoblot analysis with antibodies against collagen I, collagen IV (A). The cell lysate was also subject to immunoblot analysis with antibodies against collagen I (B), collagen IV (C), α-SMA (D) and tubulin. Representative immunoblots from three independent experiments are shown. Each bar represents the mean ± S.E. of three independent experiments. *P<0.05 versus the TGF-β1 group. C: control, T: TGF-β1, A: AICAR.

To analyze the possible intracellular signaling pathways involved in the antifibrotic effect of AICAR *in* *vitro*, fibroblast-myofibroblast transformation of the rat renal fibroblast cell line, NRK-49F was induced by TGF-β1 treatment. The production of type I and type IV collagens and α-SMA in NRK-49F cells was significantly increased in response to TGF-β1 (1 ng/mL) stimulation. Pre-treatment of NRK-49F cells with AICAR for 30 mins resulted in a dose-dependent inhibition of α-SMA and type I and type IV collagen expression both in the culture supernatant and cell lysate of NRK-49F cells after TGF-β1 stimulation (Fig. 4). The MTT test showed that cell viability was not significantly affected by the tested doses of AICAR (data not shown). Therefore, the results demonstrated that AICAR inhibits TGF-β1induced fibroblast-myofibroblast transformation.

### AMPKα1 silencing and compound C blocks the effects of AICAR on TGF-β1-induced myofibroblast activation

**Figure 5:**
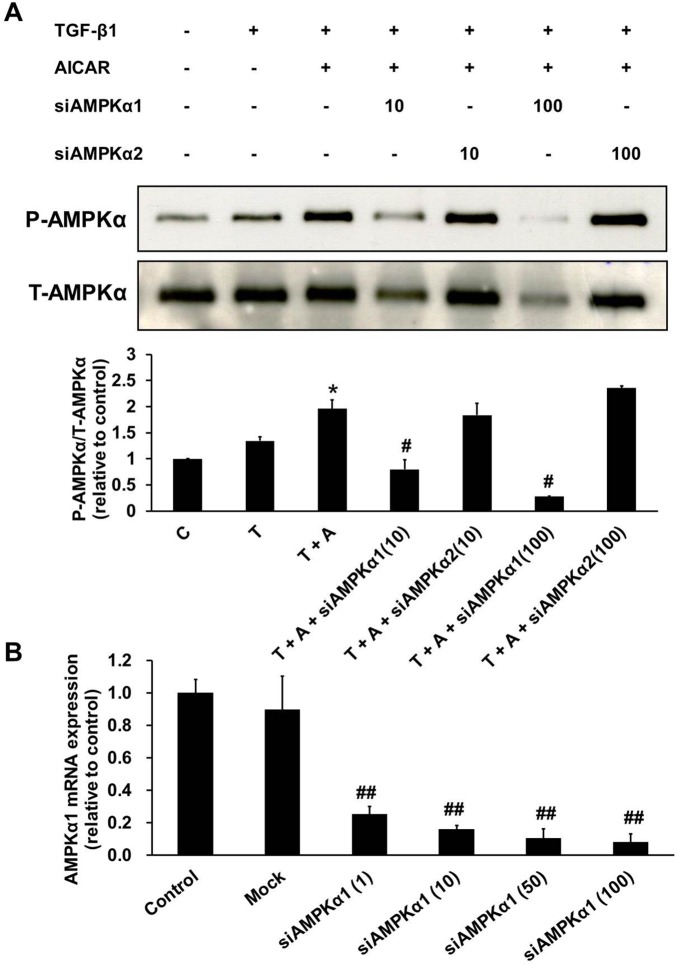
The AMPKα1 subunit is the target of the inhibitory effects of AICAR in TGF-β1-stimulated NRK-49F cells. Cultured NRK-49F cells were transfected with specific siRNA for AMPKα1 (10 nM, 100 nM) or AMPKα2 (10 nM, 100 nM) or a control siRNA for 24 h. After 24 h of incubation, transfected cells were pre-incubated with or without AICAR (0.5 mM) for 30 mins. Then, these cells were stimulated with TGF-β1 (1 ng/mL) for 24 h before harvesting. (A) The inhibitory effects of siAMPKα1 were evaluated by measuring phospho-AMPKα and total-AMPKα levels. Representative immunoblots from three independent experiments are shown. (B) The efficiency of different concentrations of siAMPKα1 (1 nM, 10 nM, 50 nM, 100 nM) on AMPKα1 mRNA expression were evaluated. Data are expressed relative to the expression in control cells without transfection. Each bar represents the mean ± S.E. of three independent experiments. *P<0.05 versus the TGF-β1 group; #*P*<0.05 versus the TGF-β1+ AICAR group; ##*P*<0.05 versus the control group. C: control, T: TGF-β1, A: AICAR.

**Figure 6:**
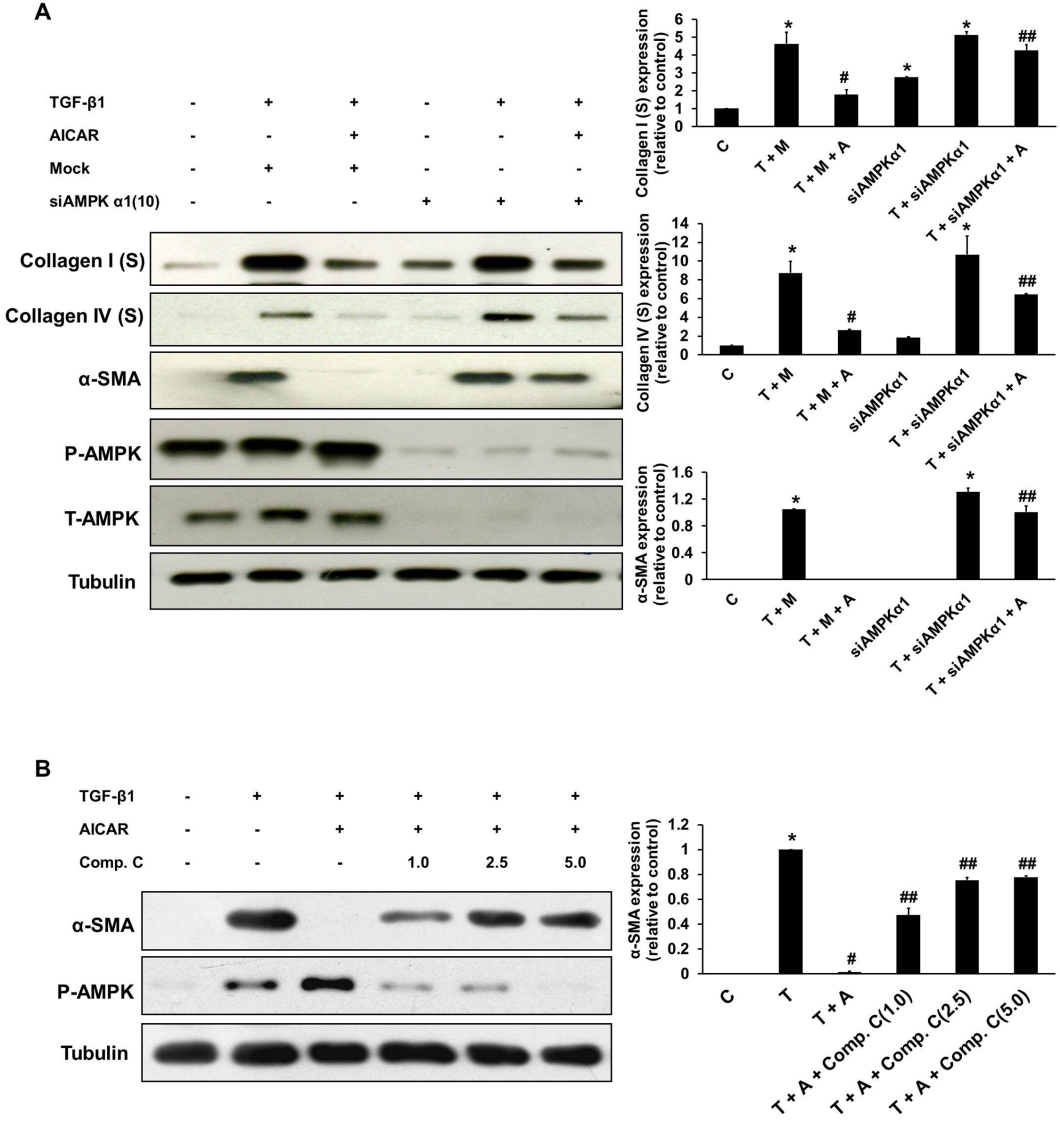
AMPKα1 silencing and compound C blocks the inhibitory effects of AICAR on TGF-β1-induced myofibroblast activation. Cultured NRK-49F cells were transfected with a siRNA specific for AMPKα1 (10 nM) or a control siRNA for 24 h. After 24 h of incubation, the transfected cells were pre-incubated with or without AICAR (0.5 mM) for 30 mins. Then, these cells were stimulated with TGF-β1 (1 ng/mL) for 24 h before harvesting. (A) Culture supernatant and cell lysates were subject to immunoblot analysis with antibodies against collagen I, collagen IV, α-SMA, and tubulin. (B) Cultured NRK-49F cells were pre-incubated with compound C (1.0 mM, 2.5 mM, or 5.0 mM) for 30 mins. Then, these cells were stimulated with TGF-β1 (1 ng/mL) for 24 h before harvesting. Immunoblotting of the cell lysates, showed that the inhibitory effects of AICAR on α-SMA expression were decreased by the treatment with Compound C in a dose-dependent manner. Representative immunoblots from three experiments are shown. Each bar represents the mean ± S.E. of three independent experiments. *P<0.05 versus the control group; #*P*<0.05 versus the TGF-β1 (+/− mock) group; ##*P*<0.05 versus the TGF-β1 (+/− mock) + AICAR group. C: control, T: TGF-β1, M: mock, A: AICAR.

To confirm the role of AICAR-mediated AMPK subunit activation in the inhibition of kidney myofibroblast activation, cells were transfected with a specific siRNAs against AMPKα1 and AMPKα2 and a control siRNA (mock). After 24 h of incubation, transfected cells were activated with TGF-β1 or/and AICAR (0.5 mM), and AMPK activation was analyzed by western blot analysis. Compared with the control siRNA, AMPKα1 siRNA, but not AMPKα2 siRNA, significantly and dose dependently inhibited AICAR-mediated AMPK activation in NRK-49F cells (Fig. 5A). We further evaluated the mRNA expression of AMPKα1 to determine the efficiency of siAMPKα1 knockdown at different concentrations (Fig. 5B). siAMPKα1 at 10 nM suppressed the expression of AMPKα1 mRNA by more than 80%. Therefore, we further examined the inhibitory effects of AICAR on extracellular matrix production after NRK-49F cells were transfected with 10 nM AMPKα1 siRNA. In comparison with the control siRNA, the AMPKα1 siRNA significantly blocked the inhibitory effects of AICAR on the production of type I and type IV collagens and α-SMA in NRK-49F cells in response to TGF-β1 stimulation (Fig. 6A). Pre-incubation with the AMPK-specific inhibitor compound C also dose-dependently blocked the inhibitory effects of AICAR on α-SMA expression in NRK-49F cells stimulated with TGF-β1 (Fig. 6B). These results revealed that the inhibitory effect of AICAR on TGF-β1-induced fibroblast-myofibrobalst transformation is partially mediated through the activation of AMPKα1.

### The effects of AICAR of the TGF-β1 signaling pathway were associated with down-regulation of ERK 1/2

**Figure 7:**
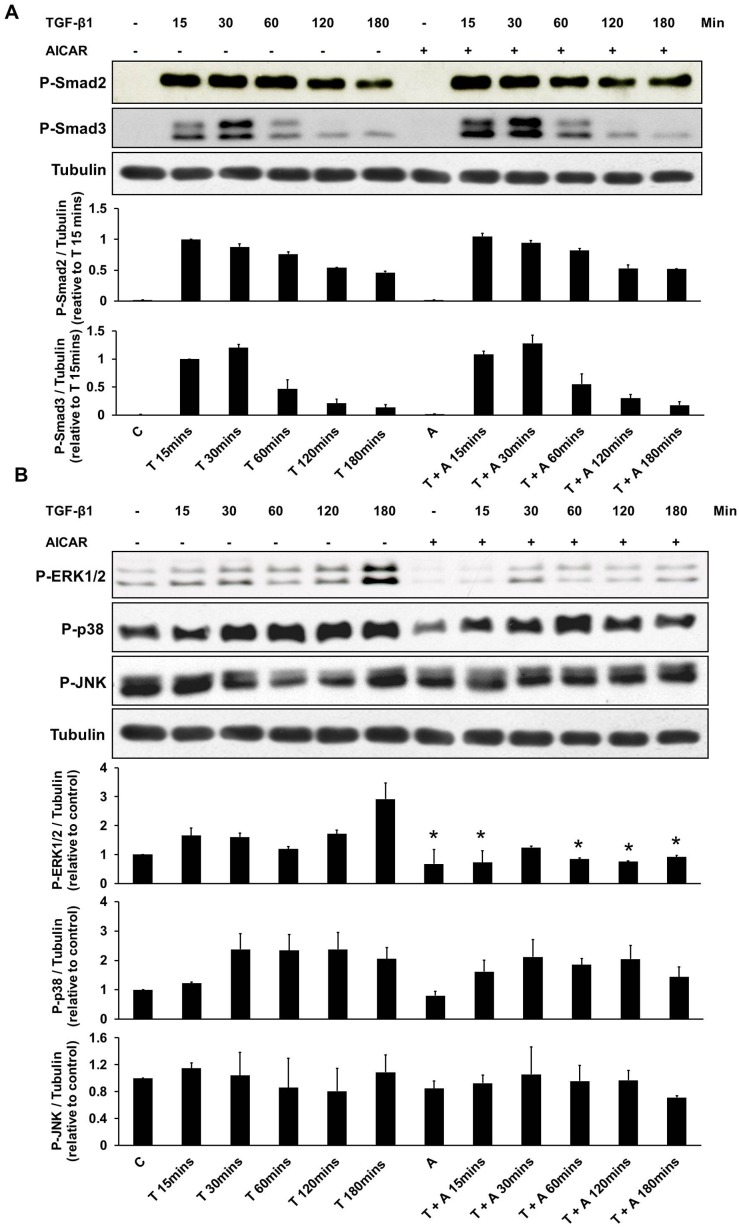
The effects of AICAR in the non-Smad TGF-β pathway were associated with down-regulation of ERK 1/2. Cultured NRK-49F cells were incubated with TGF-β1 (1 ng/mL) for 15 mins–180 mins in the presence or absence of AICAR (0.5 mM). (A) Cell lysates were subject to immunoblot analysis with antibodies against phospho-Smad2 (P-Smad2), phospho-Smad3 (P-Smad3), and tubulin. (B) Cell lysates were also subject to immunoblot analysis with antibodies against phospho-ERK1/2 (P-ERK1/2), phospho-p38 (P-p38), phospho-JNK (P-JNK), and tubulin. Representative immunoblots from three experiments are shown. Each bar represents the mean ± S.E. of three independent experiments. *P<0.05 versus the corresponding group (control or TGF-β1) at the same time duration after TGF-β1 treatment. C: control, T: TGF-β1, A: AICAR.

The effects of AICAR on the TGF-β1 signaling pathway were further evaluated. Western blot analysis demonstrated that AICAR treatment did not affect the TGF-β1-stimulated phosphorylation of Smad2 and Smad3 in the canonical TGF-β/Smads pathway in NRK-49F cells (Fig. 7A). However, ERK 1/2 activation in NRK-49F cells treated with TGF-β1 was time-dependently suppressed by AICAR treatment, whereas activation of the P38 and JNK pathway was not affected (Fig. 7B).

### The inhibitory effects of AICAR on kidney myofibroblast activation by TGF-β1 were associated with down-regulation of STAT3

**Figure 8:**
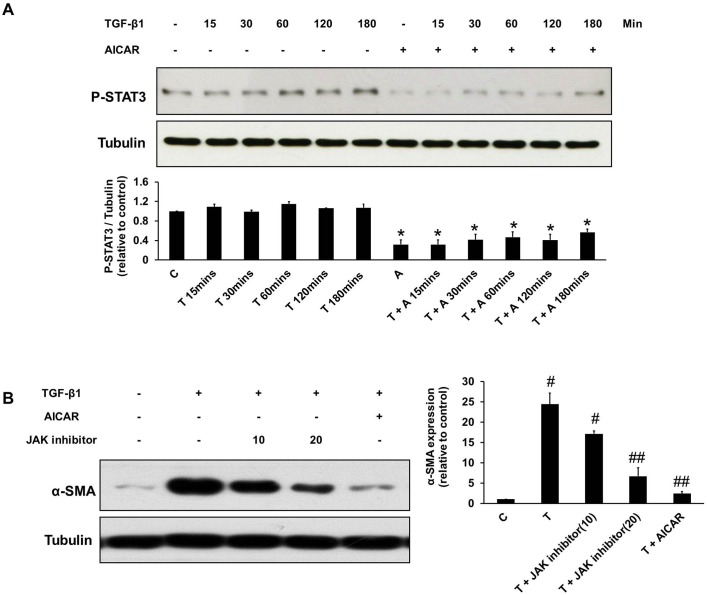
The effects of AICAR on the inhibition TGF-β1-mediated kidney myofibroblast activation were associated with STAT3 down-regulation. Cultured NRK-49F cells were incubated with TGF-β1 (1 ng/mL) for 15 mins–180 mins in the presence or absence of AICAR (0.5 mM). (A) Cell lysates were subject to immunoblot analysis with antibodies against phospho-STAT3 (P-STAT3) and tubulin. (B) TGF-β1-induced α-smooth muscle actin (α-SMA) expression in NRK-49F cells is inhibited by JAK inhibitor and AICAR. Representative immunoblots from three experiments are shown. Each bar represents the mean ± S.E. of three independent experiments. *P<0.05 versus the corresponding group (control or TGF-β1) at the same time duration after TGF-β1 treatment; #*P*<0.05 versus the control group; ##*P*<0.05 versus the TGF-β1 group. C: control, T: TGF-β1, A: AICAR.

In a previous study, AMPK activation attenuating leptin-mediated hepatic fibrosis is associated with inhibition of STAT3 activation [34]. In addition, STAT3 activation has been reported to affect numerous growth factors and cytokines, including TGF-β1 [35]. TGF-β1 time-dependently activated STAT3 phosphorylation in NRK-49F cells and pretreatment with AICAR suppressed STAT3 activation induced by TGF-β1 (Fig. 8A). Similar to the effect of AICAR, suppression of STAT3 phosphorylation by a specific STAT3 inhibitor (JAK inhibitor) also inhibited TGF-β1-induced fibroblast-myofibroblast transformation (Fig. 8B).

## Discussion and Conclusions

Tubulointerstitial renal fibrosis is the final common pathway of chronic renal diseases. Activation of myofibroblasts by TGF-β1 leads to ECM production, followed by progressive fibrosis and tubular atrophy [36]. Therefore, inhibition of TGF-β1-induced fibroblast-myofibroblast activation is a therapeutic strategy for the suppression of renal fibrosis. This study first demonstrated that AICAR treatment attenuated tubulointerstitial fibrosis in the obstructed kidney in UUO model mice. In addition, the inhibitory effect of AICAR on TGF-β1-induced fibroblast-myofibroblast transformation may be mediated through AMPK activation and down-regulation of ERK 1/2 and STAT3 activation by TGF-β1.

The AMPK pathway has been implicated in the development of liver fibrosis. Activation of AMPK by high-molecular-mass adiponectin and AICAR has been shown to inhibit the proliferation of and collagen I production by hepatic stellate cells *in* *vitro* [21]. In human primary mesangial cells, AMPK activation inhibits TGF-β1induced transcription downstream of Smad3 phosphorylation and nuclear translocation and negatively regulates mesangial-myofibroblast transdifferentiation [29]. Recently, in human proximal tubular cell (HK-2 cell), AMPK activation was also shown to inhibits TGF-β-, angiotensin II-, aldosterone-, high glucose-, and albumin-induced epithelial-mesenchymal transition [37]. In addition, in rats with subtotal nephrectomy, induction of AMPK activity by treatment with either metformin or AICAR corrects kidney metabolic disturbances and ameliorates kidney fibrosis [31]. However, whether the possible underlying mechanisms are regulated by AMPK activation is unknown and *in* *vivo* evidence for renal fibrosis inhibition in a UUO model are still lacking.

At 7 days after ureteral ligation in the present study, the obstructed kidney showed the typical features of the UUO model, and our study showed that the major marker of myofibroblast activation, α-SMA expression, was significantly reduced in the UUO kidney in AICAR-treated mice compared to that in saline-treated mice. Moreover, immunohistochemistry and western blotting analysis showed that the expression of fibrotic markers such as collagen I and fibronectin in UUO kidney were all attenuated in AICAR-treated UUO mice compared with those in saline-treated UUO mice. In addition, AMPK activation by AICAR has also been shown to exert anti-inflammatory effects, such as decreased nuclear translocation of nuclear factor κB (NF-κB) [38], inhibition of proinflammatory cytokines expression in experimental encephalomyelitis [39], and lipopolysaccharide (LPS)-induced lung injury [40]. Compatible with these previous results, our experimental results also demonstrated that AICAR treatment had anti-inflammatory effects in UUO kidney, as evidenced by the decreased the mRNA expression of TNF-α and MCP-1. However, it is difficult to understand that whether AICAR action may be upstream or downstream of TGF-β1 signaling pathways by *in* *vivo* study, we further used NRK-49F cells as *in* *vitro* model to study that how AICAR affects the pathways.

AICAR dose-dependently inhibited the production of collagen I, collagen IV, and α-SMA in the TGF-β1-stimulated renal fibroblast-myofibroblast transformation of NRK-49F cells. Both siAMPKα1 and compound C considerably abrogated the inhibitory effects of AICAR on collagen I and α-SMA production after TGF-β1 stimulation. The results indicated that the inhibitory effect of AICAR on myofibroblast activation is mostly mediated by AMPKα1 activation. In support of our findings, Lee et al. reported that siAMPK and compound C blocked the inhibitory effects of AICAR on albumin induced epithelial-mesenchymal transition in HK-2 cell, which suggested the effects of AICAR were mediated by a process involving AMPK [37]. However, the collagen I and collagen IV expression was not fully rescued in the TGF-β1+siAMPKα1+AICAR group in our study. Recent studies of AICAR also showed AMPK independent mechanisms in cell cycle regulation [41], glucose production in the liver [42], embryonic fibroblast apoptosis [28], and heat-induced sudden death [43]. Therefore, the effect of AMPK-independent mechanism for AICAR in the inhibition of TGF-β1-induced activation of kidney myofibroblasts cannot be excluded from our study.

In our study, AICAR did not affect Smad2 and Smad3 phosphorylation in the canonical TGF-β/Smads pathways. In agreement with a previous report, AMPK activation by AICAR has no effect on Smad3 phosphorylation, but only regulates Smad3 transcriptional activity in human mesangial cells [29]. In addition to the TGF-β/Smads signaling pathway, activation of the MAPK signaling pathway also plays an important role in TGF-β1-mediated renal fibrosis [44]. TGF-β1-induced myofibroblast activation involves a signaling cascade through the ERK1/2 [45], p38 [46] and JNK [47] MAPK pathways. Activation of ERK1/2 and p38 in turn induces the expression of TGF-β1, which induces a vicious cycle of progressive renal fibrosis [48]. In our study, ERK1/2 activation by TGF-β1 was suppressed in AICAR pretreated NRK-49F cells, whereas p38 and JNK activation were not affected. The results showed that AICAR-mediated inhibition of TGF-β1induced myofibroblast activation is associated with ERK1/2 inhibition.

STAT3 activation has been shown to be induced by various growth factors and cytokines such as TGF-β1 [35], platelet-derived growth factor [49] and IL-6 [50], that contribute to the development of renal fibrosis. A previous report demonstrated that adiponectin induced AMPK activation inhibiting leptin-mediated hepatic fibrosis is associated with down-regulation of STAT3 activation [34]. In a rat UUO model, increased STAT3 activation through tyrosine 705 phosphorylation in renal fibroblast has been observed [51]. Furthermore, JAK/STAT3 inhibition has been shown to ameliorate renal fibrosis *in* *vivo* [52]. Based on our results, AICAR suppressed the phosphorylation of STAT3 after TGF-β1 stimulation in NRK-49F cells. A specific STAT inhibitor (JAK inhibitor) also inhibited α-SMA expression in kidney myofibroblasts after TGF-β1 stimulation. Therefore, the inhibitory effect of AICAR on TGF-β1-induced fibroblast-myofibroblast transformation may partially mediated by STAT3 inhibition.

In conclusion, we demonstrated that AICAR reduces tubulointerstitial fibrosis in UUO mice *in* *vivo* and inhibits TGF-β1induced kidney myofibroblast activation *in* *vitro*. These effects of AICAR on the inhibition of TGF-β1-induced fibroblast-myofibroblast transformation are mediated at least in part, by activation of the AMPK pathway and decreased ERK1/2 and STAT3 phosphorylation after TGF-β1 stimulation. This study provides encouraging evidence for the therapeutic potential of AICAR, and is indicative of the possible application of AICAR for the treatment of renal tubulointerstitial fibrosis.

## Supporting Information

Figure S1 **AICAR treatment increased the levels of AMPK phosphorylation in mice treated with AICAR.** A unilateral ureteral obstruction (UUO) model was induced in adult male BALB/c mice. Sham animals had their kidney exposed but the ureter was not tied. Mice with UUO were administered intra-peritoneal AICAR (500 mg·kg-1·day) or saline 1 day before the UUO surgery and daily thereafter. Obstructed kidneys were harvested 7 days after surgery. Kidney tissue lysates were subjected to immunoblot analysis with specific antibodies against phospho-AMPK (P-AMPK) and AMPK (T-AMPK). Protein expression levels of P-AMPK were quantified by densitometry, and normalized to T-AMPK levels. Each bar represents the mean ± S.E. (n = 6 in each group). *P<0.05 between the UUO and UUO + AICAR group.
(TIF)
