# Supplementary figures and images for: The AMPK Agonist AICAR Inhibits TGF-β1 Induced Activation of Kidney Myofibroblasts
Source: PLoS One. 2014 Sep 4;9(9):e106554. doi: 10.1371/journal.pone.0106554 (PMC4154690; doi:10.1371/journal.pone.0106554)

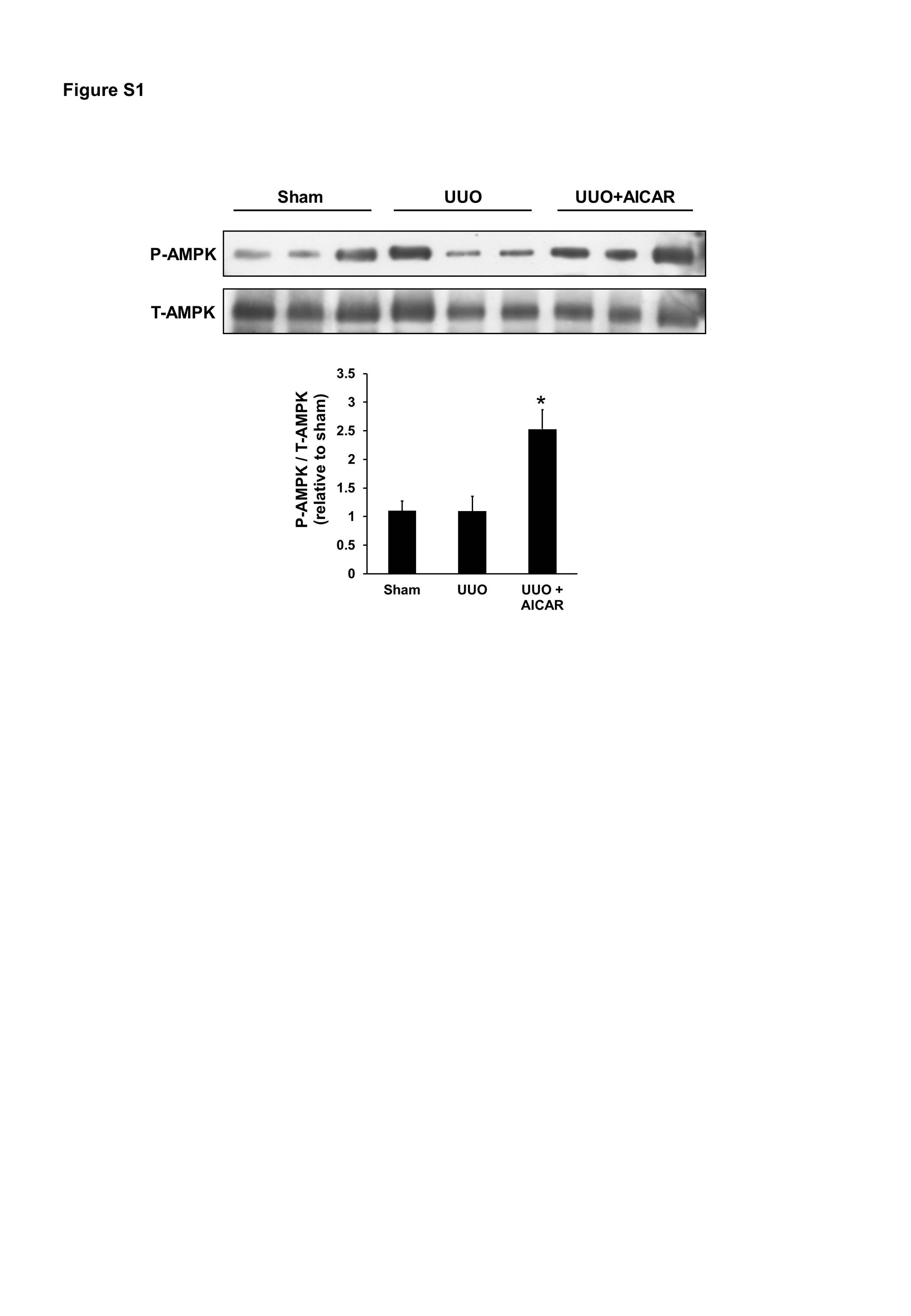

Supplement: Figure S1 — AICAR treatment increased the levels of AMPK phosphorylation in mice treated with AICAR. A unilateral ureteral obstruction (UUO) model was induced in adult male BALB/c mice. Sham animals had their kidney exposed but the ureter was not tied. Mice with UUO were administered intra-peritoneal AICAR (500 mg·kg-1·day) or saline 1 day before the UUO surgery and daily thereafter. Obstructed kidneys were harvested 7 days after surgery. Kidney tissue lysates were subjected to immunoblot analysis with specific antibodies against phospho-AMPK (P-AMPK) and AMPK (T-AMPK). Protein expression levels of P-AMPK were quantified by densitometry, and normalized to T-AMPK levels. Each bar represents the mean ± S.E. (n = 6 in each group). *P<0.05 between the UUO and UUO + AICAR group. (TIF) [file pone.0106554.s001.tif]
